# Criminal Lunatics

**Published:** 1851-10-01

**Authors:** 


### CRIMINAL LUNATICS.

The present condition of the lunatics termed "*Criminal*," confined in various asylums in this country, and the importance and necessity for the establishment of a central asylum exclusively for the reception of this class of the insane, are subjects, we are glad to perceive, at this moment attracting a large amount of professional and public attention.

In this number of our journal we have only space for the subjoined selections from the public and medical press. The following is a copy of the petition agreed to by the visiting magistrates of the Lunatic Asylum for the county of Somerset, at their meeting of the 24th of July, 1851:—

"That your petitioner has been requested by the aforesaid visiting justices to represent to your right honourable House the great importance of providing a separate place of detention for criminal lunatics, and other persons confined for purposes of justice. That the visiting justices consider that it is most important to keep the separation distinct between a lunatic asylum and a prison. That greater liberty is both possible and expedient for persons confined with a view to their cure, than could be afforded to those whose detention is demanded by justice; inasmuch as with the former their sense of responsibility is strengthened by partial liberty, and on this sense of responsibility most of the hopes of their cure depend. That the detention in asylums of persons acquitted on the grounds of insanity (many of whom are no longer insane, but must still be detained in a hopeless perpetual imprisonment) operates most unfavourably by driving them to desperation, and to acts most injurious to those confined with them, as well as to the discipline of the establishment, while the presence of such persons, and conversations with them on the subject of their trials, naturally supply to the other classes of lunatics who look forward to their release, an argument for their security hereafter, most prejudicial to public morals and their own good, inasmuch as they see clearly that whatever crimes they may commit, they will be sure, in consequence of their previous lunacy, of a verdict of acquittal, on the plea of insanity.

"That the visiting justices have been informed that a separate place of detention has been provided for such persons in Ireland. Your petitioner therefore prays that your right honourable House will take this deeply-important subject into its consideration, and make such a provision for a remedy as to your consideration and wisdom may seem meet.

(Signed) "H. W. BURNARD."

In referring to the above petition, the *Lancet* observes—

"We are rejoiced to perceive that public attention is being directed to this most important subject. The present mode of treating criminal lunatics is disgraceful to a civilized and Christian community. We do not wish to express any maudlin sympathy for crime or criminals, but if a man, after going through the ordeal of a trial for the violation of the laws of his country, is acquitted on the ground that he was not a responsible agent; that he could not distinguish between right and wrong; that he was insane, or in plainer words, that he, at the time the offence was committed, laboured under the effects of diseased brain disordering his mental faculties; then we say such a man is entitled to our warmest sympathies, and ought invariably to be treated with great kindness and consideration. The law, in its wisdom, declares that the insane ought not to be subjected to punishment, and yet as soon as a prisoner is acquitted on the plea of lunacy, he is consigned for life to the cheerless, desolate, and heart-breaking atmosphere of a public asylum, and thus subjected to the worst description of human punishment. The man in whose behalf no such plea is urged, is, in many instances, in a much more favourable position. He is accused of committing an act termed criminal. He is tried and found guilty, and perhaps either transported for a term of years, or confined in one of the county prisons for a short period; whilst the poor lunatic is incarcerated at her Majesty's pleasure—that is, for life, in a lunatic asylum, and compelled to herd day and night with the maniac. It is a monstrous perversion of language to deny that this treatment is *no* punishment. It is punishment, and that too of a severe kind! It will be admitted that there is a certain class of criminal lunatics who require to be carefully watched, and to be under surveillance for the remainder of their lives; but other cases occur in which crime is committed during a temporary paroxysm of insanity, and where recovery—complete, undeniable restoration to health—occurs either immediately after the act is perpetrated, or after a short period of confinement. But, taking the more extreme kind of case, we maintain that the law never contemplated the subjection of the criminal lunatic to the severe and protracted punishment now inflicted upon him. Insanity is considered to be a *disease* rendering the party irresponsible. The criminal placed, on this ground, beyond the pale of the law, is supposed to have self-control completely destroyed; to be as incapable of preventing himself from committing a breach of the law, as a patient is to arrest the act of vomiting after the administration of a potent emetic! What would be said if we subjected the poor man with an irritable stomach to corporeal punishment? and yet such treatment would not be more irrational and unscientific than to punish with great severity, or even at all, the unhappy lunatic guilty of criminal conduct.

"We are now talking of positive and undeniable insanity, not that pseudo-morbid condition of mind in which the person has sufficient capacity to distinguish right from wrong, and is to all intents and purposes a responsible agent.

"The reader may be under the impression that in some cases Government would be disposed, under peculiar circumstances, to relax the severity of the system, and release, when it is proved that it could be done with safety, some of the criminal inmates of Bethlem and other asylums, after their having been subjected to a long, painful, and protracted confinement. But such, alas! is not the fact. At the recent meeting of the Association of Medical Officers of Hospitals for the Insane, Dr. Forbes Winslow, when speaking of the necessity of effecting some alteration in the treatment of criminal lunatics, quoted, among other illustrations of the present conduct of the Government, the following case:—

"Dr. Winslow was consulted by the wife of Captain Johnston, who was tried for the murder of the crew of the *Tory*, and acquitted (without medical evidence) on the ground of insanity. He was accordingly sent to Bethlem. It appeared by the evidence that the murderous act was committed during a paroxysm of delirium tremens. As the medical authorities of Bethlem Hospital assured Mrs. Johnston that her husband, whatever might have been his previous state, was then perfectly sane, she presented a petition to Government for his liberation, and offered, in the event of his being released, to provide an ample and sufficient guarantee that Captain Johnston should be taken out of the country into a far distant land, and that he never should set foot on English soil again. The petition was rejected, and the poor broken-hearted wife informed that there was no chance of her husband ever gazing on the blue sky of heaven, beyond the gloomy courts of the asylum into which he was transferred after his acquittal on the plea of mental derangement.

"Without entering into the merits of this particular case, it may be asked, is this justice? Is this common humanity? Do the authorities at the Home Office consider it a part of their creed that patients never recover from attacks of homicidal insanity, and if evidence, clear and undoubted, of recovery presents itself, that no relaxation is to take place in the treatment of the unhappy prisoners? If this be their doctrine, the sooner its hollowness, its absurdity, its cruelty, are exposed, the better for the credit of a Christian community and a Christian government. The criminal lunatic, we again maintain, is in many instances in a worse condition than the felon consigned to one of the model prisons, or even to the hulks or penal settlements. The man confined in Pentonville or Reading prison has advantages and enjoyments which M'Naughten, Oxford, Tuckitt, Johnston, and others, confined in Bethlem, are strangers to. The felon transported to one of the penal settlements can, if he is well-conducted, obtain, after a short term of punishment, a ticket of leave, which amounts almost to entire liberation. He may then enter the service of the colonists, and perhaps ultimately obtain a free pardon; but alas! what hope is held out to the lunatic, who, in a moment of transient madness and irresponsibility, under the affliction of disease, commits an offence against the State, be it murder, manslaughter, theft, or arson? Well might the words said by Dante to be written at the entrance of the infernal regions, be inscribed over the portals of that portion of Bethlem Hospital appropriated to the poor criminal lunatic:

'Voi che entrate lasciate ogni speranza.'

"It is to be hoped that the day is not far distant when the Government will take more enlightened views on these and kindred topics, and that the opinions of men of experience, judgment, and knowledge, connected with our own profession, will exercise a right influence on the discussions of those into whose hands are entrusted grave and heavy responsibilities. As to the necessity of having separate asylums for the reception of criminal lunatics there cannot be a doubt, and asylums too in which the poor unhappy inmates will have within their reach more of the comforts and enjoyments of life. Some change must soon take place. Let us hope that a brighter and a happier morn will soon dawn upon the poor, wretched, criminal lunatics at present confined within the iron gratings of Bethlem and other public lunatic asylums; and that if the patients are not fitted for entire liberation, their relatives will be permitted to transfer them to a private establishment where greater comfort and relaxation, with the same degree of security, are to be obtained."

The following observations, in connexion with the same subject, appeared in the columns of the *Morning Chronicle* newspaper, a journal ever ready to advocate an enlarged, liberal, humane, and philosophical view of all questions involving the well-being of the state, and the happiness of the people.

The remarks we are about to quote proceed from the pen of Mr. F. H. Dickenson of Kingsweston.

"I wish you would call the attention of the public to the neglect of the government in not providing a separate place of confinement for lunatics, and persons supposed to be lunatics, who are detained for the purposes of justice. Such a prison has been established in Ireland, and the urgent necessity of providing one here has been repeatedly pressed on the government by the Commissioners in Lunacy, and the managers of the Lancashire and other asylums. But while the Government and the House of Commons have wasted their time on legislation, trifling if it be not tyrannical, they have none, it would seem, for matters that are not pressed on public attention by urgent and influential agitators. There may possibly be objections, from the time it would take, to that reform of the law of lunacy which is called for, and which is recommended by the commissioners; but I am at a loss to see the difficulty in obtaining a grant of the House of Commons for establishing a prison for the due custody of a class of persons who are already a heavy charge on the country in another way. It implies no real increase of expense beyond the outlay for building, and it could not long occupy the time of the House of Commons. Only let those who have the public charge of lunatics, and who therefore feel the evil, knock loudly at the door of the Home Office, and a willing assent will be yielded to Lord Shaftesbury when he brings the matter before the next session of parliament.

"The commissioners state as follows, in their report for 1850, pages 16, 17:—

'We entertain the same opinions which we expressed in our last report relative to the class of insane patients termed state or criminal lunatics; for although the arrangement, to which we referred last year, with the proprietor of Fisherton House, near Salisbury, for receiving harmless criminal lunatics, has been carried out, the small number so received gives very inadequate relief to the asylums in which (exclusive of more than 100 criminal patients in Bethlem Hospital) 264 of such patients were confined on the 1st of January last.

"Your lordship is aware that the construction of lunatic asylums is so essentially different from that of prisons, that an effectual security against the escape of criminals cannot be provided without restricting the liberty of other patients with whom they are necessarily associated, and materially interfering with that treatment and general arrangement which ought to be adopted for their benefit. Criminal patients have, therefore, escaped, and must continue to escape from asylums and houses licensed for the reception of the insane. As an instance of this we may mention the fact which was brought by us specially under the notice of Secretary Sir G. Grey, that a most active and cunning criminal patient escaped for the fifth time from Hoxton House, in February last. [He has since escaped a sixth time; his name is Henry Adams].

"'Our objection applies especially to such lunatics as have been charged with the more heinous offences; and it has been frequently brought under our notice by the friends and relatives of patients, and also the patients themselves, when conscious of their being associated with criminal lunatics, have considered such association as a great and unnecessary aggravation of their calamity.'

"There was held in London on the 17th of July a meeting of the 'Association of Medical Officers of Hospitals for the Insane.' You have been a little disposed to quiz, lately, the annual meetings for objects of all imaginable kinds that are now held, and certainly some of them are odd enough. But the periodical meetings of those who have a common want or a common interest, are a necessary and most useful consequence of our present state of social freedom; and whether they merely become acquainted with each other and amuse themselves, or help forward a great cause, or strengthen the foundation and extend the knowledge of some science, or make themselves ridiculous, we may tolerate the absurdity for the much greater amount of substantial good that is obtained.

"The meeting then comprised many gentlemen who have devoted themselves to the care of public asylums in the central and southern parts of England, and one even from Belfast. The principal business was this very question of the treatment of criminal lunatics, and they agreed to petition the Government 'for the establishment of an institution apart from Bethlem, exclusively for the reception of persons acquitted of crime on the plea of insanity.' The unfortunate, desolate, and unhappy condition of the criminal lunatics was feelingly dwelt on by all the speakers, and it appeared to be the unanimous opinion of the Association that sufficient attention had not been paid to their state. It was thought that great good would result from the separation of the criminal from the other lunatics confined in Bethlem and other public asylums.

"They also suggested amendments in the laws relating to county asylums, which I hope they will take means to make public, and Dr. Winslow and four others were appointed a committee to report on this matter, in all its branches, including the law of the property of lunatics. In all this they are but seconding the commissioners, who have expressed their wish, and appear to have devised plans, for similar improvements in the law.

"Those whose experience justifies their doing so, may, I apprehend, suggest modes for the classification and separate detention of different classes of lunatics, from which their more sure recovery or greater comfort may be secured. I will not attempt to trespass on this extensive and somewhat delicate ground. And on the point which I have brought before you I will merely venture to point out, in addition to the remarks I have quoted, that it is important to keep clear the distinction between a *Prison* and an *Asylum*—between a place of confinement and disgrace, and one where these things ought to be studiously avoided; in order that the poor man may, by a system of relief and comfort, be aided to bring the better feelings of his nature to bear on the mental disease which has for a time incapacitated him from society and his usual work. The commissioners, in the passage of their Report which I have quoted, have glanced at the importance of giving as much liberty as possible to patients. It is plain that a system of trust and confidence, which is so salutary to strengthen the feeling of responsibility and moral rectitude in those who are recovering, must be interfered with when the restraint necessary for criminals is used. The discontent arising from confinement is also obvious and most prejudicial. Among those whom one calls, for the sake of convenience, but incorrectly, criminal lunatics, there are—besides those who, having no control over their own actions, would by every one be considered innocent—many others who committed crime while temporarily insane, or who simulated madness. I suppose there is no question but that all these persons—when the acts they have committed would, if done by sane persons, have been murder or any other of the more heinous crimes—ought to be confined for life. Consider, then, the feelings of the man during his lucid intervals, or of him who knows he has never been insane at all—but who each of them know that they will never be let out, and have free scope to employ all the energy that villany and despair can give them, to the prejudice of their companions in a county asylum. And the latter are naturally led to expect, from seeing sane persons in confinement along with them, that their own *imprisonment* may be unduly prolonged; and a feeling must occasionally force itself on their minds, that if ever they get out and commit crimes, even the most heinous, their punishment cannot be worse than what they are then enduring. Many think that the law of acquittal on account of lunacy is, in its present state, most injurious to society. It is not my business to offer any opinion on that, but every one must agree with me that a system which familiarizes every pauper lunatic in the country with the idea of the boundless privilege of doing wrong, which he seems to enjoy under that law, ought at once to be abolished; and this can only be effected by the separate confinement of these very different classes of men."

